# Effective elimination of lead from polluted wastewater utilizing a novel nanocomposite derived from byproducts of drinking water industry

**DOI:** 10.1186/s13065-025-01549-4

**Published:** 2025-07-03

**Authors:** Elsayed A. Elkhatib, Mohamed L. Moharem, Ahmed F. Saad, Safa Abdelhamed

**Affiliations:** 1 Department of Soil and Water Sciences, Faculty of Agriculture (El‑Shatby), Alexandria University, Alexandria, 21545 Egypt; 2 Regional Center for Food and Feed, Agricultural Research Center, Alexandria, Egypt

**Keywords:** Water treatment sludge, Adsorption equilibrium, Kinetics models, Recyclability, Polluted water

## Abstract

Industrial and consumer waste can introduce trace metals into a water supply, resulting in detrimental effects on both human health and natural ecosystems. The primary objective of this research is to produce and characterize a new nanocomposite sorbent (Zeolite- nano-drinking water treatment residuals, Ze-nWTR), followed by assessing its effectiveness in removing Pb(II) from wastewater under varying conditions such as solution pH, presence of competing ions, sorbent quantity, temperature, and contact time using batch experiments. The Langmuir and power function models were utilized to accurately depict the Pb (II) adsorption isotherm and kinetic data. The nanocomposite exhibited a maximum adsorption capacity (q_max_) of 198.7 mgg^−1^, which was determined to be 2.6 and 5.5 times greater than the adsorption capacities of nWTR (75 mgg^−1^) and Zeolite (36 mgg^−1^), respectively. Significant increase in sorbed Pb(II) was noticed above the point of zero charge (pH_zpc_) value of nanocomposite (7.2). Thermodynamic experiments demonstrated that Pb adsorption process onto nanocomposite was endothermic and involuntary. The supposed mechanism of Pb (II) sorption onto nanocomposite was discussed. The final results indicated that nanocomposite (Ze-nWTR) can likely apply for Pb(II) removal from wastewater due to its high affinity towards Pb adsorption, repeatedly used, economically and sustainability.

**Supplementary Information:**

The online version contains supplementary material available at 10.1186/s13065-025-01549-4.

## Introduction

The contamination of water by trace metals is considered a major global issue due to its detrimental impact on human health and ecosystems [1]. Lead with unknown biological action in living organisms could be responsible for sundry sorts of metabolic and physiological disorders in humans, animals and plants [2]. Lead exists in many industries such as, paint, battery, steel, smelting, electroplating, pesticides, and inorganic fertilizers [3–6]. This toxic metal can reach human body through contaminated water; therefore, decrease Pb level in wastewater is needful to avoid its reverse effects.

Currently, the widespread removal techniques of trace metals from wastewater are electrodialysis, sulfide/flocculation precipitation, ion exchange, membrane separation, and adsorption [7–10]. However, adsorption has confirmed to outperform the other approaches due to low price, high performance and reused of adsorbent [11–14]. Therefore, it is largely important to advance low price and highly effective adsorbents for metal removal from wastewater [7, 15–17]. Zeolites are a group of chemically related mineral substances that contain mainly hydrated aluminum and silicon compounds; these minerals have largely studied due to its low price and high retention efficiency for trace metals [18, 19]. Likewise, water treatment residuals (WTR) that are by-products of the coagulation and flocculation phase of the drinking water treatment process daily produce huge amounts of waste/residue known as water treatment sludge (WTS) during the purifying of drinking water. These waste materials underwent a transformation into nanomaterials via high-energy ball milling [20]. Numerous studies have validated their effectiveness as superior adsorbents for various trace metals found in contaminated water and soil [21–23].

The use of composites in wastewater treatment has been gaining importance in recent years due to its chemical stability and improved affinity towards trace metals [24–26]. Yang et al. [27] reported that zeolite-geo-polymer composites prepared from industrial solid waste have a high Pb(II) removal capacity from solution which refer to its high surface area and hierarchical pore structure. In addition, results obtained from Medykowska et al. [28] study demonstrated the excellent adsorption capacity of zeolite carbon composites towards trace metals with higher tendency towards lead. Consequently, the modification of zeolite can easily take place to enhance its capacity for adsorbing trace metals [29–31]. However, to the best of the authors' knowledge, there have been no publications discussing the use of a Zeolite-Nano Drinking Water Treatment Residuals (Ze-nWTR) nanocomposite for Pb(II) removal. This research introduces a novel composite sorbent—Ze-nWTR—by combining zeolite with nano-sized drinking water treatment residuals (nWTR) to create a highly efficient material for Pb(II) removal. The study investigates the effectiveness of Ze-nWTR under various conditions, including solution pH, competing ions, sorbent dosage, temperature, and contact time, using batch experiments. Moreover, the removal mechanism of Pb(II) by the Ze-nWTR nanocomposite is explored, and the composite's efficiency in removing lead from real wastewater is evaluated through column experiments. This approach offers a new and innovative solution for enhanced Pb(II) remediation from contaminated water.

## Materials and methods

### Preparation of Ze–nWTR nanocomposite

The WTR was obtained from the water treatment plant in Kafr El-Dawar, El-bohera, Egypt. The Table (S1) displays the physical and chemical attributes of the WTRs. The samples of WTR were air dried, ground, and then passed through sieves with pore diameters of 2 mm and 51 μm. The WTRs were subsequently processed into nanoparticles following the technique established by Elkhatib et al. [20]. The zeolite utilized was procured from Delta Biotec Company situated in Borg Alarab, Egypt, with a comprehensive analysis provided in Table (S2).The samples of zeolite were dried for a period of 24 h at a temperature of 80 °C, pulverized, and sifted through a 100 mesh screen prior to utilization. The composites were produced in the subsequent manner: 1.0 g of Zeolite samples were exposed to 0.1 M NaNO_3_ for duration of one week to accomplish homoionization, and subsequently dried. The clay and nWTR (w/w) were mixed in ratios of 2:1. The mixture underwent ultrasound treatment for thirty minutes, followed by vigorous stirring for 120 min at room temperature. The solid underwent five washings with a 1:1 mixture of ethanol and water, followed by drying and storage.

### Characterization

The crystallographic properties of the materials were determined using X-ray diffraction (XRD) on a D/Max-2500 diffractometer (Rigaku, Japan) employing Cu Kα radiation (λ = 0.15418 Å) at 40 kV and 30 mA. Scans were conducted over a 2θ range of 15°–80° at a rate of 8° min⁻^1^. Surface morphology and elemental composition were examined using scanning electron microscopy (SEM; FEI Quanta 200, USA) equipped with energy-dispersive X-ray spectroscopy (EDX), operated at an accelerating voltage of 15 kV. The specific surface area, pore volume, and pore size distribution were analyzed via nitrogen adsorption–desorption isotherms using a BET surface area analyzer (Quantachrome Instruments, USA) following degassing at 150 °C for 12 h under vacuum [32]. Functional groups and surface chemistry were identified by Fourier transform infrared spectroscopy (FTIR; Nicolet iS5, Thermo Fisher Scientific, USA) within the wavenumber range of 4000–400 cm⁻^1^, using 32 scans per sample at a resolution of 4 cm⁻^1^.

### Sorption kinetics of Pb(II)

Sorption kinetic experiments were performed utilizing 50 mL polyethylene centrifuge tubes. In these experiments, 0.2 g of each sorbent under investigation was introduced into 20 mL of Pb(II) solution with an initial concentration of 500 mg/L. The mixtures were placed on a horizontal shaker (Biobase, Cole-Parmer) operating at a speed of 170 rpm and a temperature of 25 °C for varying durations between 5 and 1500 min. The pH was kept constant by automatic titration with HCl or NaOH. During each time interval, samples were collected, centrifuged at 4000 rpm for 10 min, and subsequently passed through a 0.45 μm Millipore filter. An atomic absorption spectrometer (contrAA 300) was utilized to determine concentration of Pb (II) in the filtered solution. Various kinetic models were utilized to examine the acquired kinetic data (Table S4- Supplementary). The percentage of Pb(II) removed was calculated using the following equation:

$$\text{\%}\hspace{0.17em}\text{Removal}=\left(\frac{Ci-Ce}{Ci}\right)*100\%$$

where Ci and Ce are the initial and equilibrium of Pb(II) concentrations (mg/L) respectively.

The selected Pb(II) concentration of 500 mg/L reflects levels found in severely contaminated industrial and mining effluents, such as those discharged from battery manufacturing, metal plating, and pigment production industries [33, 34]. Previous studies have reported Pb(II) concentrations in real wastewater reaching up to 1000 mg/L [35], especially in unregulated or legacy contamination sites. Therefore, this study simulates worst-case scenarios, ensuring that the tested adsorbent's performance is assessed under highly challenging conditions. Additionally, evaluating the performance of adsorbents at these levels is crucial for several reasons: (1) It aids in the design of emergency response systems for industrial spills or historical pollution sites, (2) It allows for the testing of adsorbent capacity limits under challenging conditions, offering valuable insights into their scalability and durability, (3) It informs the pre-treatment requirements necessary before discharge into municipal treatment facilities or for reuse in irrigation, and (4) Adsorbents that demonstrate high efficacy at elevated concentrations can also be remarkably effective at lower, more common contamination levels, thus proving their versatility in addressing both point-source and diffuse pollution challenges.

### Adsorption isotherms

Adsorption equilibrium tests were conducting by introducing 0.2 g of each sorbent studied into a 20 mL Pb^2+^ solution in a form of lead acetate -Pb(CH_3_COO)^2−^—with concentrations ranging from 40 to 800 mg L^−1^. After subjecting the solution to agitation at 170 rpm and 25 °C for duration of 20 h, samples were collected and underwent a series of processes including centrifugation and filtration. The resulting solution was then analyzed to determine the total quantity of Pb, following the procedures outlined earlier. The sorption data obtained was subsequently examined using the 6 models listed in Table (S5- supplementary). To determine the thermodynamic parameters of the Ze-nWTR composite, the Arrhenius equation was utilized, incorporating sorption data collected at temperatures of 287, 297, and 307 K, across 3 pH levels (4, 7, and 9), and 4 concentrations of Pb(II). A comparable series of adsorption experiments was conducted to evaluate the influence of competing cations on the adsorption of Pb by Ze-nWTR. The experiments were carried out with the existence of 3 competing cations (Ni, Zn, Cu) at Pb (II) concentration of 500 mg L^−1^. In addition, we performed similar adsorption tests by employing 3 distinct quantities of each sorbent (0.1, 0.2, and 0.3) to investigate the impact of sorbent dosage on Pb(II) removal.

### Thermodynamic study

The influence of temperature on Pb (II) adsorption process was investigated by calculating the thermodynamic parameters ΔG° (J mol^−1^), ΔH° (J mol^−1^), and ΔS° (J mol^−1^ K^−1^). The supplementary materials provide a detailed explanation of the determination and computation of these parameters.

### Reusability study

The reusability of the Ze-nWTR nano-composite was assessed to determine its effectiveness. The nano-composite containing Pb was dried and placed in 50 mL of 0.01 M HCl. Subsequently, it was agitated for 120 min at ambient temperature. The resulting mixtures were filtered, dried, and the rejuvenated nanocomposites were utilized again for Pb(II) removal. This adsorption–desorption process was replicated and evaluated over a span of six cycles.

### Efficiency of nanocomposite for Pb adsorptive removal

#### Bach experiment

The effectiveness of the nano-composite (Ze-nWTR) on Pb(II) removal was assessed through a batch experiment utilizing actual wastewater samples obtained from the discharge of Rakta paper manufacturing plant and the Al-Bilali agricultural drainage. The chemical composition of the wastewater samples studied are presented in Table (S3).

#### Column experiment

To assess the effectiveness of the Ze-nWTR nanocomposite in removing Pb(II) from real wastewater, column filtration experiments were performed using a down-flow setup. PVC columns (20 cm in height and 2.5 cm internal diameter) were packed with a homogeneous mixture of Ze-nWTR and sand at a defined mass ratio (Fig. S1). A peristaltic pump was used to maintain a constant flow rate, delivering Pb(II)-contaminated solution from a reservoir to the top of the column. Effluent samples were collected at regular time intervals for analysis.

##### Role of sand

*Sand was incorporated exclusively as a passive support medium* to ensure uniform packing, enhance permeability, and minimize clogging during continuous flow. This approach is consistent with standard practices in fixed-bed adsorption systems, where inert materials like sand or gravel are commonly used to maintain column integrity and flow uniformity without participating in the adsorption process [6, 36]. Accordingly, *the contribution of sand to Pb(II) removal is considered negligible*, and the adsorption performance reported in this study is attributed entirely to the Ze-nWTR nanocomposite. This methodological design allows for a realistic simulation of field conditions while ensuring that the sorbent’s efficiency is accurately evaluated in a dynamic system.

## Results and discussion

### Surface properties of the sorbents

#### nWTR

The surface morphology of the WTR nanoparticles (nWTRs) was examined through SEM analysis to assess surface characteristics, particles arrangement, and the alterations that occurred following Pb(II) adsorption. The SEM images depicting nWTRs prior to and following Pb(II) adsorption are illustrated in Fig. (S2). Prior and after Pb(II) adsorption, the SEM images reveal that the nWTRs exhibit a spherical shape, with particle sizes fall within the nanoscale range (Fig.S2 above and middle). Following Pb(II) adsorption, the SEM image (Fig. S2 middle) displays a layer of adsorbed Pb(II) coating the surface. The SEM–EDX elemental analysis indicated that nWTR contains Fe, Si, and Al in 49.57, 21.77, and 6.3% of the overall elemental contents respectively. Furthermore, XRD analysis confirmed that nWTR components are mostly aluminum and silicon oxides and amorphous iron prevail with no existent of crystalline iron-Al (hydr) oxides (Fig. (S2-bottom). The X-ray diffraction pattern ascertained that amorphous iron aluminum (hydr)oxides, and silicon oxide dominated all nWTR, with no apparent crystalline iron–Al(hydr)oxides. The abundant iron and aluminum in water treatment residual nanoparticles could have great influence on Pb(II) adsorption by nWTRs.

#### Zeolite

**Fig. 1:**
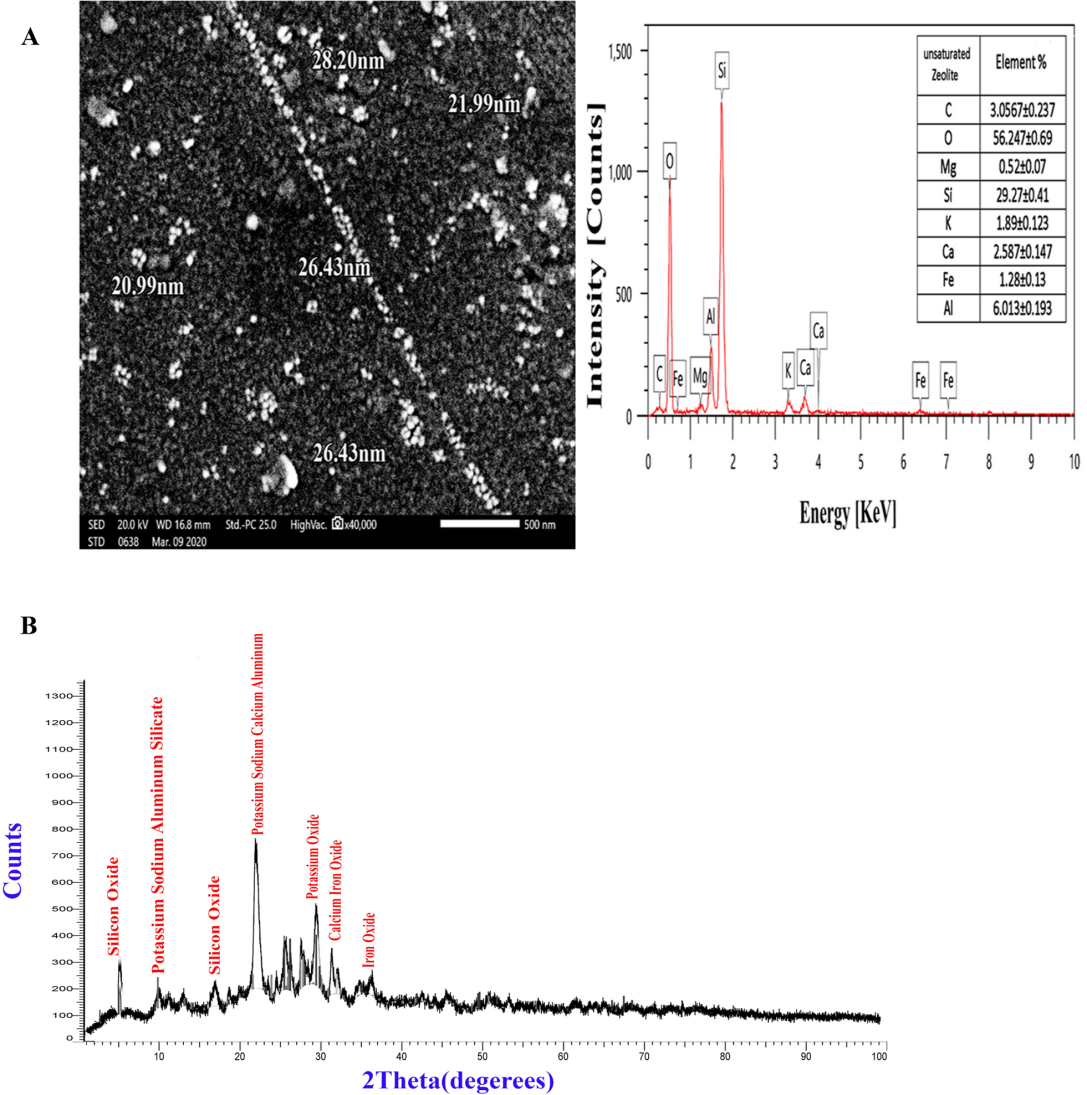
SEM image and EDX spectrum (**A**) along with XRD analyses of Zeolite (**B**)

The scanning electron microscopy (SEM) image shown in Fig. 1A reveals that the majority of zeolite particles have a spherical shape, with sizes ranging between 20 and 28 nm. Elemental analysis using SEM–EDX indicates that the zeolite is predominantly composed of oxygen, silicon, aluminum, and iron, which together make up 91% of the total elemental content. The remaining 9% consists of carbon, calcium, potassium, iron, and magnesium. The X-ray diffraction (XRD) patterns in Fig. 1B show a prominent peak at 2θ = 4.5 and 67.861, confirming a significant presence of silicon oxide (SiO2) in the zeolite sample. Additional peaks observed at 2θ values of 9.4 and 25 suggest the presence of KNaAlSiO_4_, while peaks at 2θ values of (31.8 & 40), (35.021 & 46.5), and (41.9 & 76.5) correspond to the identification of CaFe_2_O_4_, Fe_2_O_3_, and K₂O, respectively.

#### Ze-nWTR nanocomposite

**Fig. 2:**
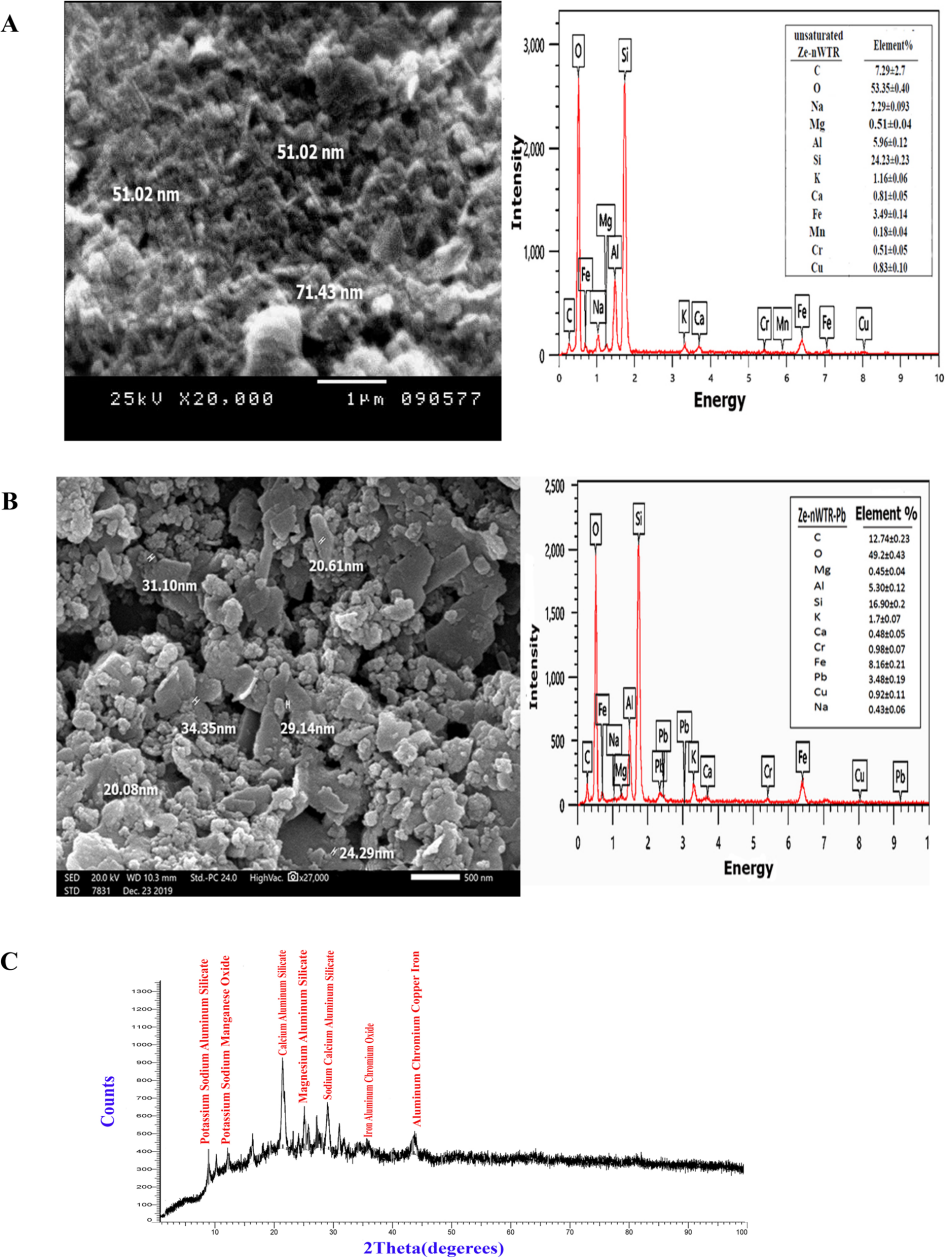
SEM image and EDX spectrum of nanocomposite (**A**); Pb loaded Ze-nWTR nanocomposite (**B**) and XRD analyses of Ze-nWTR nanocomposite (**C**)

Figure 2A shows the surface morphology of the nanocomposite, where zeolite wafers are clearly visible with spherical nWTR nanoparticles distributed across their flat surface. These spherical nanoparticles range in size from 51.02 to 71.43 nm, falling within the nano scale. The zeolite clay present on the surface effectively inhibits the agglomeration of these nanoparticles, while its spherical particles contribute to enhanced stability and facilitate easier handling. According to EDX analysis, the main elements present in the nanocomposite are oxygen, silicon, carbon, aluminum, iron, potassium, and sodium, accounting for roughly 98% of the total elemental composition. The remaining 2% contains calcium, magnesium, copper, and chromium, with their concentrations ranging from 0.53% to 83%. The presence of aluminum and iron suggests that nWTR may enhance the adsorption capacity of the Ze-nWTR nanocomposite for lead (Pb).

The SEM/EDX analysis of the Pb(II)-loaded Ze-nWTR nanocomposite, shown in Fig. 2A, reveals that after Pb saturation, the individual particle sizes range between 20.08 and 31.10 nm. EDX elemental analysis of the Pb-saturated Ze-nWTR composite confirms the presence of a lead peak (3.48%) among the detected elements (Fig. 2B).

In Fig. 2C, the XRD pattern of the nanocomposite clearly displays peaks at 2θ = 9.48 and 12.81, indicating the presence of K-NaAlSi_3_O_8_ in the sample. Additional peaks at 2θ values of 12.7 and 28.6 suggest the presence of NaKMnO_4_, while peaks at 2θ values of 22 and 27.7 correspond to CaSi_2_O_8_ and Al_2_Si_2_O_8_. The identification of iron, aluminum, chromium oxide (Cr_2_O_3_), and various metal ions, including aluminum, chromium, iron, and copper, was also confirmed (Fig. 2C).

### Specific surface area

The specific surface area (SSA) of nWTR, Zeolite, and Ze-nWTR composite were determined. nWTR exhibited a significantly higher SSA of 129 m2 g^−1^ compared to Zeolite with 39 m^2^g^−1^ and Ze-nWTR nanocomposite with 89 m^2^ g^−1^. The elevated SSA of nWTR indicates its potential to provide additional adsorption sites for Pb on the surface of the nanocomposite.

### FTIR spectroscopy

**Fig. 3:**
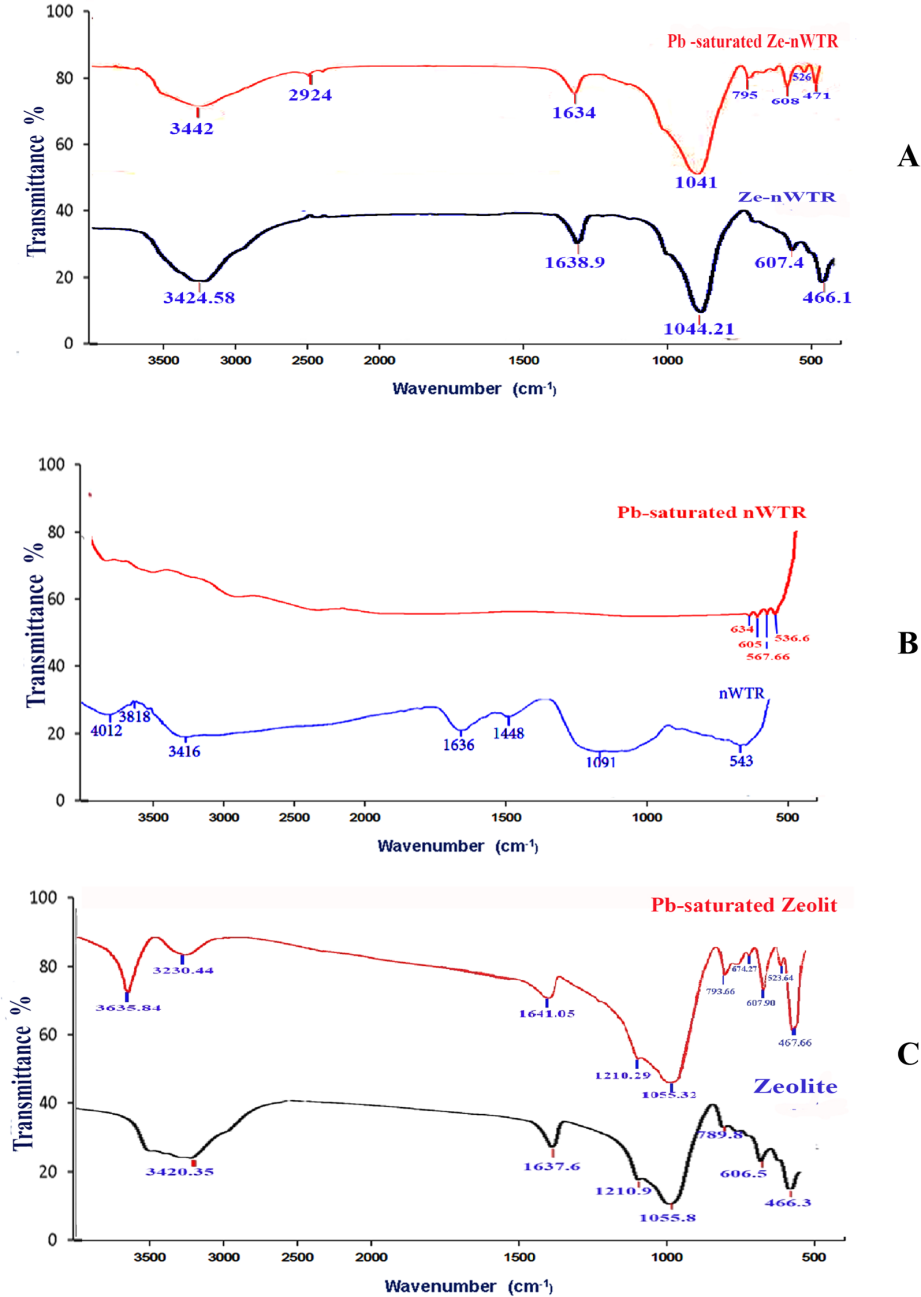
FTIR spectra of Ze-nWTR (**a**), nWTR (**b**) and Zeolite (**c**) Prior to and following Pb(II) loading

The FTIR spectrum of Ze-nWTR (at wavenumber range from 3500 to 500 cm^−1^) (Fig. 3) displayed a significant wide peak at 3424 cm^−1^, which corresponds to the bending vibrations of O–H bonds. Additionally, a smaller peak at 1638 cm^−1^ was observed, indicating the bending mode of the H–O–H molecule [37]. Furthermore, a significant band at 1044 cm^−1^ was identified as the FeOH vibration of feroxyhdoxide, while a band at 466 cm^−1^ was associated with the stretching vibration of O–AL–O [38]. The presence of Pb on the surface of Ze-nWTR led to notable changes in the spectral profile. The band at 3424 cm^−1^ (O–H bending vibration) shifted to the location of 3442 cm^−1^ which confirmed the partnership of OH group on Ze-nWTR surface in Pb sorption process. Meanwhile, the bands' intensity has increased, leading to a shift in their positions from 1638.9 cm^−1^ to 1634 cm^−1^ (O–H bending vibrations), from 1044 cm^−1^ to 1041 cm^−1^, and from 607 cm^−1^ to 608 cm^−1^ (O–AL–O stretching vibration). Furthermore, two new bands associated with d-FeOOH have emerged at 471 cm^−1^ [39]. The alterations in both position and intensity of these bands clearly indicate a distinct molecular interaction. Hence, the nanocomposite suggests collaboration between OH, O–Al-O, and FeOOH entities in the adsorption process of Pb. The presence of Pb on the surface of nWTR caused notable alterations in the spectral characteristics, as depicted in Fig. 3b. The 4012 cm^−1^ band (O–H bending vibration) has completely stopped, providing further evidence of the significant involvement of the surface OH group of nWTR. Additionally, the disappearance of the bands at 3416, 1636, and 1091 cm^−1^, as well as the shift of the band at 3420 cm^−1^ to 3635 cm^−1^ (O–H bending vibration) on the surface of zeolite, indicate the involvement of various entities such as O–Al–O, C–Br, and FeOOH in the Pb adsorption process by nWTR. This retention of Pb (II) on the Zeolite surface demonstrates the participation of the surface hydroxyl group of Zeolite in the Pb(II) retention process, as shown in Fig. 3C.

### Adsorption kinetics

**Fig. 4:**
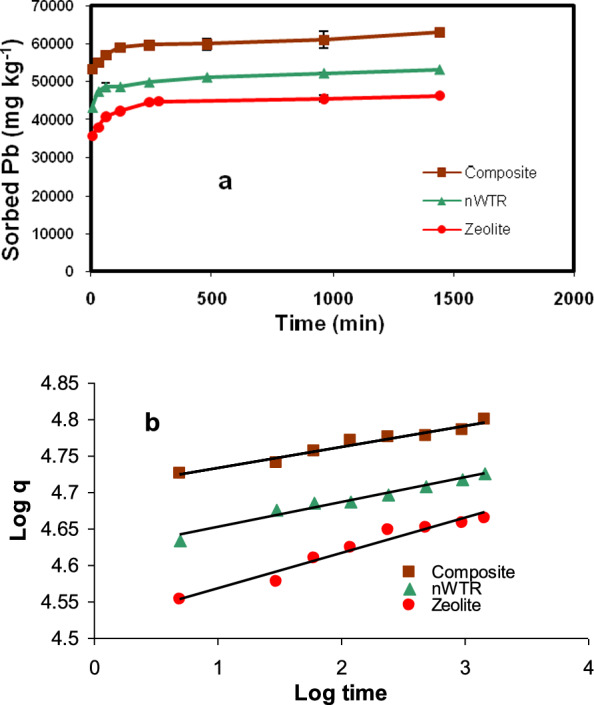
The adsorption kinetics of lead onto nWTR, Zeolite, and the nanocomposite (**a**) and linear power function model plots for lead adsorption onto the three studied sorbents (**b**)

The kinetic experiments were employed to identify time required for Pb (II) to reach equilibrium for the three adsorbents. It was noted that the time required to reach equilibrium for nanocomposite, nWTR and Zeolite were 2 h, 6 h, and 8 h, respectively. Thus, using nanocomposite accelerated Pb(II) kinetic reaction. Such data are substantial from economical point of view in wastewater treatment field [40]. Sorption kinetics of Pb by the sorbents studied displayed an forthright fast sorption by which about 90% of Pb was sorbed in the first 5 min and kept track of delayed sorption at 298 K (Fig. 4a) The fast initial adsorption of Pb(II) is a superficial occurrence and the vacancies present on the sorbent surface could be loaded speedily in the first stages and followed by a delayed emigration and spread [41].

### Kinetics modeling

Sorption kinetics of Pb (II) by the threes sorbents studied were performed and analyzed using first-order [42], Elovich [43], Intraparticle diffusion [44], and Power function [45] kinetic models. The determination of the excellent fit between the experimental data and the predicted values of the model was established through the utilization of coefficients of determination (R^2^) and the standard error of estimate (SE) values. The model parameters, R^2^and SE values of Pb adsorption onto the three sorbents studied are illustrated in Table 1. The prosperity of the model to depict the kinetics of Pb (II) sorption accordant to a comparatively high R^2^ value nearby or equal to 1 and low SE value. Power function equation has been affirmed to be the best kinetic equations studied competent to characterize Pb(II) sorption kinetics by nWTR, Zeolite and Ze-nWTR nanocomposite as evidenced by the overall high R^2^ (0.96) values and low SE (0.003763) values (Table 1 and Fig. 4b).

**Table 1:** Calculated kinetic parameters for Pb adsorption onto3 adsorbents

Models	Parameter	nWTR	Zeolite	Composite
Elovich q_t_ = (1/β) ln(α β) + (1/β) lnt	α (mg g^−1^ min^−1^)	4.06E + 102	2E + 07	8.28E + 72
	β (mg g^−1^)	0.00480	0.0005	0.00349
	R^2^	0.958	0.8524	0.86
	SE	89.52	1853.4	240
First order ln (q_ο_−q) = a−k_a_ t	K_d_ (min^−1^)	0.0067	0.015	0.013
	a (µg g^−1^)	7.0256	9.319	7.318
	R^2^	0.9481	0.935	0.954
	SE	0.3128	1.068	0.31
Parabolic diffusion q = a + k_a_t^1/2^	K_d_ (µgg^−1^ min^−1/2^)	29.52	247.2	37.4
	a (μg g^−1^)	48,250	38,901	48,600
	R^2^	0.864	0.578	0.63
	SE	160.8	3135	394.3
Power function q = *k*_*a*_ C_o_ t^1/m^	K_a_ (min^−1^)	41,476.3	33,075.01	50,652.4
	1/m	0.0342	0.0483	0.0287
	R^2^	0.9702	0.9556	0.962
	SE	0.00039	0.0099	0.001

### Adsorption isotherms

**Fig. 5:**
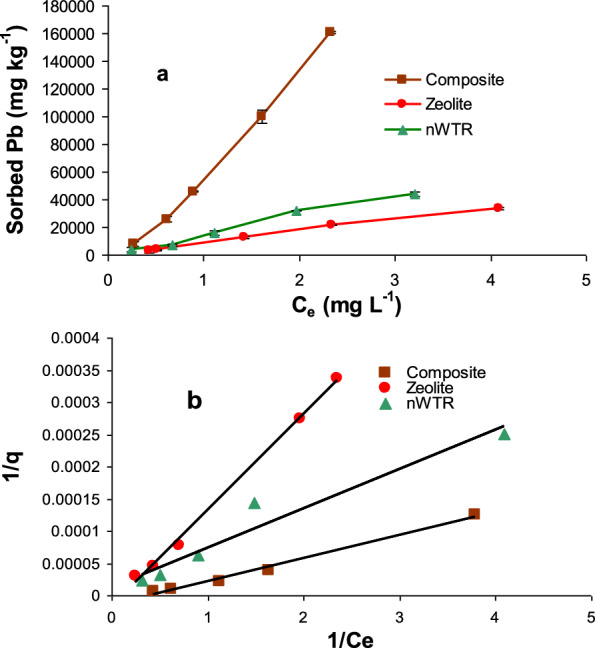
Isotherms for Pb sorption by nWTR, Zeolite and nanocomposite (Ze-nWTR) (**a**) and Langmuir isotherms for the three studied sorbents (**b**)

Lead adsorption isotherms were carried by Zeolite, nWTR and Ze- nWTR composite at 40–640 mg L^−1^ initial concentrations (Fig. 5a**)**. It is noticed that the quantity of adsorbed Pb(II) was increased as a function of Pb(II) concentration. Strong interaction between Pb and the Ze-nWTR composite adsorbent is noticed comparing with the other two sorbents; since the sorbents efficiency were in the order of nano-composite > nWTR > Zeolite. The form of Ze-nWTR composite sorption isotherm is an H-type isotherm [46] reflecting high affinity between sorbent and sorbate while the form of Pb(II) sorption isotherm of nWTR and Zeolite is L-type isotherm expressing moderate attractiveness for Pb(II) in comparison to Ze-nWTR composite.

### Adsorption equilibrium models

In order to accurately predict the parameters of sorption by nWTR, Zeolite and Ze-nWTR composite, seven sorption isotherms models were tested (Table 2). The smallest SE value and the highest R^2^ value of the equilibrium models tested are the used criteria for selection of the models that accurately represent the sorption data [47]. Based on coefficient R^2^and SE values, the experimental data for Pb adsorption onto the three sorbents fit better to Langmuir isotherm model than the other isotherm studied models (Table 2 and Fig. 5b). The maximum adsorption capacity (q_max_) value was higher for Ze-nWTR (198.7mgg^−1^) followed by nWTR (75 mgg^−1^) and Zeolite (36 mgg^−1^). Indeed, covering Zeolite with nWTR could strongly increase the capability of Zeolite for Pb (II) retention since q_max_value of Ze-nWTR was 2.6 and 5.5 times larger than q_max_ values of nWTR and Zeolite sorbents, respectively.

**Table 2:** Calculated parameters of 7isotherm models for Pb adsorption onto 3 adsorbents

Models	Parameter	nWTR	Zeolite	Nanocomposite
Freundlich q_e_ = K_F_C_e_^1/n^	*K*_*F*_ (mL g^−1^)	17,940.2	12,480	17,640
	1/n	1.047	1.111	1.212
	R^2^	0.9863	0.968	0.963
	SE	0.148	0.227	0.245
Langmuir q_e_ = q_max_(K_L_ C_e_/1 + K_L_C_e_)	q_max_ (mg g^−1^)	75	36	198.7
	K_L_ (L mg^−1^)	0.133	0.1	0.09
	R^2^	0.944	0.997	0.990
	SE	0.00002	0.00004	0.00002
Elovich q_e_/q_m_ = K_E_C_e_exp(− q_e_/q_m_)	q_max_ (μg g^−1^)	166,666	200,000	100,000
	K_E_ (L mg^−1^)	1.08	1.06	1.14
	R^2^	0.59	0.335	0.7526
	SE	0.143	0.212	0.184
Temkin θ = RT/∆Q lnK_0_C_e_	ΔQ (kJ mol^−1^)	14.4	12.60244	16.227
	*K*_0_(L g^−1^)	3.057	3.218633	2.997
	R^2^	0.775	0.846	0.786
	SE	0.106	0.11	0.077
Fowler–Guggenheim(FG) K_FG_C_e_ = θ/1−θ exp(2 θ w/RT)	*W*(kJ mol^−1^)	− 5.265	− 2.333	− 3.914
	*K*_FG_(L mg^−1^)	0.098	0.109	0.081
	R^2^	0.930	0.552	0.965
	SE	0.081	0.255	0.119
Kiselev k_1_C_e_ = θ/(1−θ)(1 + k_n_ θ)	*k*_1_(L mg^−1^)	0.108	0.085	0.08
	*kn*	2.940	6.068	4.515
	R^2^	0.989	0.866	0.978
	SE	0.172	0.3701	0.223
Hill–deBoer K_1_C_e_ = θ/(1−θ) exp(θ/(1−θ)−K_2_θ/ RT)	K_1_ (Lmg^−1^)	0.098	0.105	0.078
	K_2_ (kJ mol^−1^)	14.51	8.493	10.848
	R^2^	0.949	0.804	0.986
	SE	0.082	0.255	0.118

### Influence of operating conditions on Pb removal

#### Solution pH

**Fig. 6:**
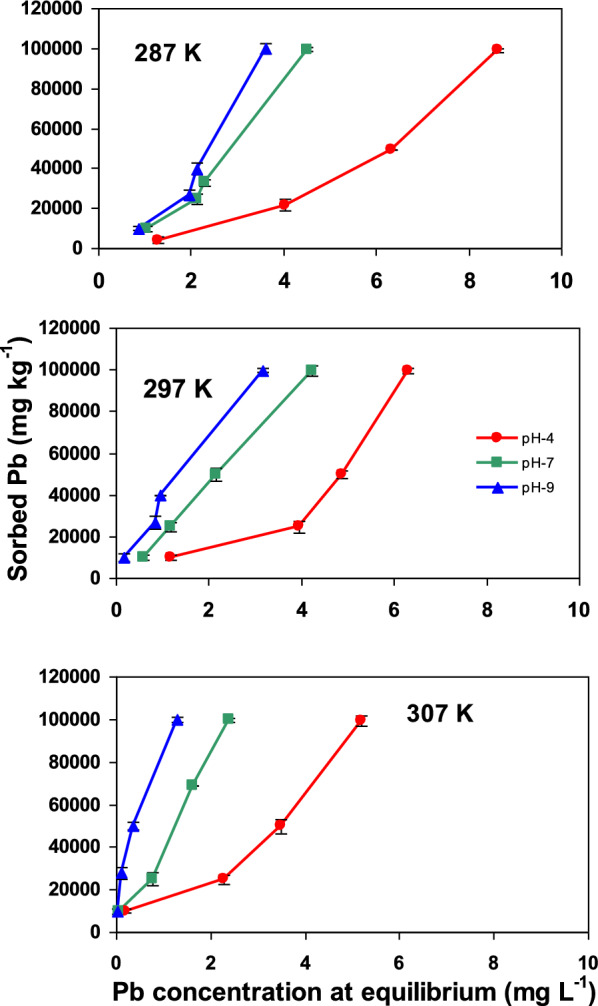
Impact of pH on Pb sorption onto Ze-nWTR nanocomposite at 3varying temperatures

Solution pH plays a critical role in controlling the adsorption process, as it influences both the surface charge of the Ze-nWTR nanocomposite and the speciation of Pb(II) in the aqueous phase [48, 49]. In this study, the impact of initial pH on Pb(II) adsorption was evaluated at three pH levels (4, 7, and 9), using a fixed sorbent dose of 0.2 g and at three temperatures (287, 297, and 307 K) (Fig. 6).The removal efficiency of Pb(II) ions increased with rising solution pH, reaching a maximum at pH 9. This trend can be attributed to the interplay between the surface charge of the sorbents and the speciation of Pb(II), both of which are strongly influenced by pH [50]. The point of zero charge (pHzpc) of the nanocomposite was determined to be 7.2 (Fig. S3).At pH values above 7.2, the surface of the nanocomposite becomes increasingly negatively charged, thereby enhancing the electrostatic attraction between the sorbent and the positively charged Pb(II) ions. This reduction in repulsive interactions facilitates greater Pb(II) adsorption. Conversely, under acidic conditions (pH < 7.2), the sorbent surface is protonated due to an abundance of H⁺ ions, leading to increased positive surface charge. This causes electrostatic repulsion between the sorbent and Pb(II) ions, thereby reducing adsorption efficiency.

#### Competing cations

**Fig. 7:**
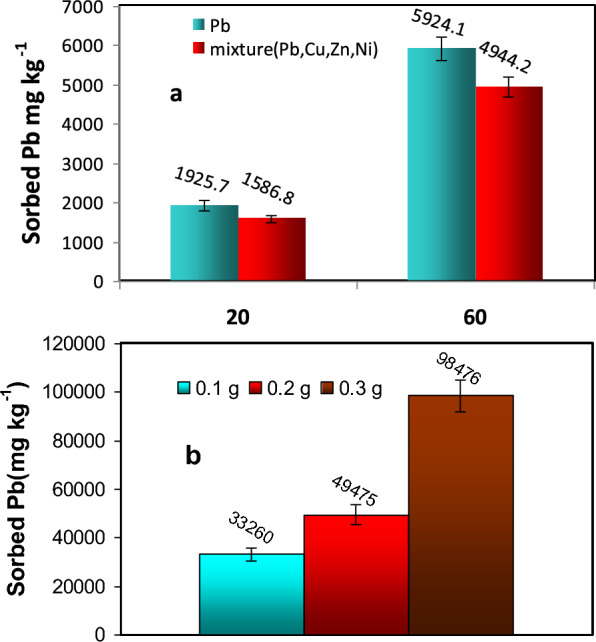
The impact of competitive cations (Ni, Zn, Cu) on Pb sorption onto Ze-nWTR at two different Pb concentrations (**a**), and3 varying sorbent doses (**b**)

Studying the competitive adsorption among trace metals onto Ze-nWTR is crucial due to the presence of different metal ions in industrial wastewater effluents that can interact with sorbents and vie for binding sites. A batch adsorption experiment was conducted with and without Zn + Cu + Ni at Pb (II) concentrations of 20 and 60 mgL^−1^ to determine the levels of Pb sorbed by Ze-nWTR in both single and multiple elements systems (Fig. 7a). The results indicated that the presence of Zn^2+^, Cu^2+^, and Ni^2+^ cations decreased the amount of Pb ions adsorbed by the nanocomposite from the solution. In the single system, Ze-nWTR removed a higher quantity of Pb(II) (1925.7 and 5924.2 mg kg^−1^) compared to the multi-element (Pb + Zn + Cu + Ni) system, where the Pb(II) removal was 1587 and 4944 mg kg^−1^ at initial Pb concentrations of 20 and 60 mgL^−1^ respectively (Fig. 7a). This can be attributed to the abundant available sorption sites on Ze-nWTR nanocomposite in comparison to those of multi-element system [51]. Furthermore, the size of ions in a multi-element system directly impacts the sorption of Pb by influencing the competition for sorption sites, ion pairing, and the availability of active sites on the sorbent. Smaller ions, like Zn^2^⁺, Cu^2^⁺, and Ni.^2^⁺, may reduce Pb sorption by occupying binding sites more readily or forming stronger complexes, demonstrating the influence of other ions on the Pb sorption process [52]

#### Adsorbent dose

The sorbent's ability to handle a specific initial concentration heavily relies on the dosage of the sorbent. An experiment was conducted to assess the impact of Ze-nWTR composite mass on the removal of Pb (II) from an aqueous solution. Various amounts of Ze-nWTR composite ranging from 0.1 to 0.3 g were added, using 20 mL of an initial Pb(II) concentration of 500 mg L^−1^, with a contact time of 60 min. Figure 7b displays the influence of the nanocomposite mass on Pb removal. The results indicate greater differences in Pb(II) sorption capacities among different doses. With increasing nanocomposite dosage from 0.1 to 0.2 and 0.3 g, the Pb removal increased from 33,260 to 49,475 and 98,476 mgkg^−1^, respectively at a fixed Pb^2+^ concentration. An increase in the mass of the sorbent results in a greater surface area, thereby increasing the number of adsorption sites available for Pb(II). This, in turn, boosts the amount of Pb that the nanocomposite can absorb as the dosage of the nanocomposite rises. Nevertheless, the rate at which metal sorption increases often diminishes as the sorbent surface approaches its maximum adsorption capacity. The degree to which the amount of sorbed metal rises with increased dosage is influenced by the sorbent's maximum adsorption capacity and the number of available binding sites, indicating that the benefits may decrease with higher dosages.

#### Temperature

The effect of temperature on the removal of Pb(II) by Ze-nWTR nanocomposite was investigated at 3 different temperatures (287, 297, and 307 K) and 4 concentrations of Pb(II) (Fig. 6). It is noticed that Pb(II) adsorption reaction increased as a results of raising temperature from 287 to 307 K. Furthermore, the Langmuir adsorption capacity of the nanocomposite increased from198.7 mgg^−1^ to 284 mgg^−1^ with the increase of the temperature from 279 to 308 K. Through chemisorption reactions, the encouragement of metals movement towards adsorbents-reactive sites occurs at higher temperatures [53].

#### Thermodynamics of Pb(II) adsorption by Ze-nWTR nanocomposite

The thermodynamic standards of Pb (II) retention onto Ze-nWTR nanocomposite were calculated to gain a comprehensive understanding of Pb sorption. The calculations were performed at four initial Pb concentrations (100, 250, 500, and 1000 mg L^−1^) and 3 pH values (4, 7, 9). The findings indicated that the ΔG° value for the sorption of Pb(II) at an initial concentration of 100 mg L^−1^ and pH 9 was determined to be − 22.723, − 26.751, and − 31.512 kJ mol^−1^ at temperatures of 14 °C, 24 °C, and 34 °C, respectively (Table 3). The escalation in negative ΔG° values with rising temperature indicates an increase in Pb adsorption at elevated temperatures, as illustrated in Table 3, implying a greater number of sorption sites at higher temperatures. Additionally, the negative ΔG° values increased due to the rise in pH values, suggesting more effective sorption as pH values increased from 4 to 9. [54–57]. Also, at different initial solution concentrations, the ΔS^°^ values were negative suggesting an associative mechanism through Pb sorption process [58]. The trend of ΔH° values increased consistently with different solution concentrations, suggesting that the retention of Pb on Ze-nWTR is an endothermic process [58] (Table 3). Moreover, as the initial concentration increased, the ΔH° values decreased, suggesting lower energy requirements for Pb sorption on Ze-nWTR at higher initial concentrations. Typically, ΔH° values falling between 2.1 and 20.9 kJ mol ^−1^ signify non-specific adsorption, whereas values fall within the range of 20.9 to 408 kJ mol^−1^ indicate chemisorption [59]. Thus, the elevated ΔH° values between 34.1 and 103 kJ mol^−1^ observed at various initial solution concentrations imply specific adsorption between Pb and the nanocomposite.

**Table 3:** Calculated thermodynamic parameters of Pb adsorption bythe nanocomposite at 3 pH values and 4 initial Pb concentrations

Initial concentration (mg l^−1^)	pH	T (K)	ΔG◦ (J mol^−1^)	ΔS◦ (Jmol^−1^ K^−1^)	ΔH◦ ( J mol^−1^)
100	4	287	− 19,638	− 241.81	49,921
		297	− 21,580		
		307	− 24,474		
	7	287	− 21,142	− 253.90	51,594
		297	− 24,076		
		307	− 26,220		
	9	287	− 22,723	− 439.45	103,520
		297	− 26,752		
		307	− 31,512		
250	4	287	− 20,553	− 219.73	42,442
		297	− 22,956		
		307	− 24,947		
	7	287	− 21,795	− 233.02	45,088
		297	− 24,101		
		307	25,230-		
	9	287	− 23,088	− 243.66	46,939
		297	− 25,235		
		307	− 27,962		
500	4	287	− 21,455	− 172.79	28,147
		297	− 23,153		
		307	− 24,910		
	7	287	− 22,132	− 197.72	34,617
		297	− 24,099		
		307	− 26,087		
	9	287	− 22,828	− 225.98	42,190
		297	− 24,603		
		307	− 27,348		
1000	4	287	− 22,541	− 197.3	34,085
		297	− 24,511		
		307	− 26,487		
	7	287	− 22,948	− 205.55	36,068
		297	− 24,934		
		307	− 27,059		
	9	287	− 23,438	− 231.77	43,031
		297	− 25,897		
		307	− 28,073		

### Mechanisms of Pb sorption

FTIR, XRD, and EDX analysis were used to interpret the mechanisms of Pb (II) sorption on Ze-nWTR nanocomposite. The EDX elemental analysis validated the Pb(II) adsorption on the surface of nanocomposite by appearance of a lead peak (3.48%) amongst the elements detected after saturation of nanocomposite with Pb (Fig. 2B). The role of OH functional group of Zeolite on Pb(II) adsorption was evident from the FTIR analysis since the band at 3420 cm^−1^ (O–H bending vibration) has been shifted to the location of 3635 cm^−1^ after Pb(II) adsorption as illustrated in Fig. 3C. Thus the OH surface group of Zeolite can attract Pb(II) to its outer surface to form outer sphere complexes by ion exchange between H^+^ related to OH group and Pb(II) [60]. The suggested adsorption mechanism is illustrated by the following equation:

$${\text{HO}} - {\text{Ze}} - {\text{OH}} + {\text{ Pb}}^{{{2} + }} = {\text{ HO}} - {\text{Ze}} - {\text{O}} - {\text{Pb }} + {\text{ 2H}}^{ + }$$

The disappearance of the bands in nWTR, at 4012, 3416, 1636, 1091 cm^−1^, 1636 cm^−1^ after Pb(II) retention according to FTIR analysis (Fig. 3B) demonstrates the significant role of nWTR-functional groups such as FeOOH on Pb adsorption. Furthermore, based on EDX elemental analysis the remarkable decrease in Fe percent of nWTR from 49.59 to 6.25 after Pb saturation reflexes the function of iron in Pb retention Fig. (S2) (Supplementary materials). Thus, it is postulated that Pb (II) incorporates into the Fe oxyhydroxide structure- nWTR through Pb solid solution formation in the Pb-Fe co-precipitate. Indeed, the larger atomic size of Pb (II) in the Fe oxyhydroxide structure as compared to Fe that causing displacing the octahedrally coordinated Pb(II) unit vertical to the sheet by 0.30 ± 0.02A ˚ from the FeO_6_ unit position can be responsible for the decrease of Fe% after Pb(II) retention onto nWTR [61]. The likely mechanism for the co-precipitation of lead and iron can be represented by the following equation:

$${\text{Fe }}\left( {{\text{OH}}} \right)_{{{3}({\text{s}})}} + {\text{ Pb}}^{{{2} + }} = {\text{ PbH}}\left( {{\text{OH}}} \right)_{{{3}({\text{s}})}} + {\text{ Fe}}^{{{3} + }}$$

where, the Pb^2+^ substitution for Fe ^3+^ is counterbalanced by H^+^ addition into the crystalline structure [62, 63]. Such observation is supported by EDX elemental analysis which demonstrated considerable increase in Fe% from 3.49% to 8.16% after Pb retention on the nanocomposite (Fig. 2A). In addition, the location shift of the band related to FeOH vibration- on Pb-saturated Ze-nWTR nanocomposite-from 1044 cm^−1^ to 1041 cm^−1^—(Fig. 3A) could be indicative of Pb–Fe co-precipitate with FeOH group onto the nanocomposite surfaces. It is therefore, suggested that Pb sorption on Ze-nWTR nanocomposite occurs at first through outer sphere surface complexation between Pb and OH function group of Zeolite. In sequence, the Pb adsorbed on zeolite can be incorporated into the Fe hydroxide structure of nWTR through Fe-Pb precipitate formation.

### Reusability

**Fig. 8:**
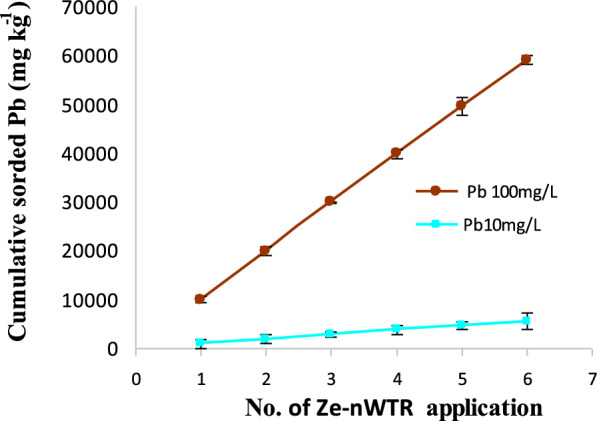
Impact of repeated application of the nanocomposite on the total amount of sorbed Pb at two initial Pb concentrations

The reusability and constancy of Ze-nWTR nanocomposite was examined for six sequential adsorption/desorption periods utilizing 0.01 M HCl solution to desorb laden Pb(II). The cumulative lead sorbed by Ze-nWTR nanocomposites at 10.0 mgL^−1^ and/or 100.0 mgL^−1^ initial Pb concentrations are illustrated in Fig. 8. The nanocomposite's ability to remove Pb(II) from aqueous solutions remained relatively unchanged after six consecutive cycles, regardless of the two different concentrations of Pb(II) used. Thus, the nanocomposite can be efficiency repeatedly used even after 6 cycles reflecting its practicability, economically and sustainability.

### Efficient removal of Pb by nanocomposite

#### Batch adsorption test

A batch experiment was carried out to evaluate the efficacy of the Ze-nWTR nanocomposite in eliminating Pb (II) from actual wastewater samples. The findings reveal that the Ze-nWTR nanocomposite achieves an impressive removal efficiency of 96.73% for industrial wastewater contaminated with lead from Rakta Company. Furthermore, when applied to al-Bilali agricultural drainage, the removal efficiency increases to 97.44%. This remarkable level of removal underscores the material's potential for treating wastewater laden with Pb(II). The results indicate that Ze-nWTR is highly effective in extracting Pb(II) from various wastewater matrices, suggesting its wide-ranging applicability. This is particularly important in light of the extensive contamination of water sources with lead due to industrial processes and agricultural runoff, positioning Ze-nWTR as a promising option for environmental remediation. Furthermore a key finding of this study is that common anions in wastewater, such as SO₄^2^⁻, CO₃^2^⁻, HCO₃⁻, and Cl⁻, did not significantly affect the Pb(II) removal efficiency of Ze-nWTR. This is important because Ze-nWTR's ability to remove Pb(II) despite the presence of these anions highlights its resilience to common pollutants, making it highly practical for real-world applications.

#### Column study

The Ze-nWTR nanocomposite demonstrated a removal efficiency of 96.3% and 97.11% for agricultural drainage and industrial discharge, respectively, under continuous flow conditions at a flow rate of 3 mL min^−1^ (Fig. S1). The high rate of Pb(II) removal achieved by the nanocomposite suggests its potential as a sorbent for removing Pb(II) from industrial and agricultural wastewater.

### Comparison of different adsorbents for Pb removal

The efficacy of employing Ze-nWTR nanocomposite for the elimination of Pb(II) from wastewater was further confirmed by comparing the maximum adsorption capacity (q_max_) of this nanocomposite with various adsorbents documented in the literature, as presented in Table 4. It is evident that the q_max_ of Ze-nWTR nanocomposite (198.7 mgg^−1^) surpasses that of other nanocomposite sorbents. These findings demonstrate the exceptional performance and efficiency of Ze-nWTR nanocomposite in the removal of Pb(II) from contaminated wastewater.

**Table 4:** Comparison between the maximum adsorption capacities (q_max)_ of Pb (II) in several studies listed in the literature

Adsorbent	q_max_ mgg^−1^	References
Graphene oxide/chitosan/FeOOH nanocomposites	111.11	[64]
β-cyclodextrin modified graphene oxide nanosheets	149.53	[65]
Iron-coated zeolite	11.16	[66]
Fe–Cu alloy coated cellulose nanocrystals	39.9	[67]
Fe_3_O_4_/C (Oleaster seeds) nanocomposite	123.5	[68]
modified corncob nanocomposite	11	[69]
nMgO-bentonite nanocomposite	75	[70]
Polyacrylamide grafted carboxymethyl cellulose with magnetic graphene oxide and N, N-methylene bis-acrylamide	86	[71]
1,4-phenylne diisocyanate (LPDIC)	20.86	[72]
Noug stalk activated carbon(NSAC)	89.25	[73]
Ze-nWTR nanocomposite	198.7	Current study

## Conclusions

The synthesis of Ze-nWTR nanocomposite was attained and investigated using SEM–EDX, XRD and FTIR analyses. The SEM picture of prepared Ze-nWTRs demonstrated that spherical shaped nWTR nanoparticles in 51.02–71.43 nm range are spread on Zeolite wafers. The optimum conditions of Pb elimination by the nanocomposite are at pH 9 and 307 K. Additionally, rising the reaction temperature from 287 to 307 K, increased Langmuir adsorption capacity of the nanocomposite (q_max_) from 198.7 mgg^−1^ to 284 mgg^−1^. The nanocomposite accelerated Pb(II) kinetic sorption reaction. The conceivable mechanisms of Pb adsorption on the nanocomposite comprised outer sphere surface complexation and Fe-Pb co-precipitation. The powerful of Ze-nWTR nanocomposite in removing Pb(II) from real wastewater under continuous flow conditions was evident since 96.3%, and 97.11% of Pb (II) was removed from agricultural drainage and industrial discharge, respectively. The lead that was incorporated into the Ze-nWTR nanocomposite demonstrated significant resistance to Pb (II) release over six consecutive adsorption/desorption cycles, indicating the robust stability of Pb(II) within the nanocomposite. The conclusive findings suggest that the Ze-nWTR nanocomposite has the potential to serve as an excellent sorbent for the removal of Pb(II) from industrial wastewater.

## Supplementary Information

Supplementary Material 1.

## Data Availability

All data generated or analyzed during this study are included in this published article and its supplementary information files.
