# Supplementary material for: Effective elimination of lead from polluted wastewater utilizing a novel nanocomposite derived from byproducts of drinking water industry
Source: BMC Chem. 2025 Jul 3;19(1):188. doi: 10.1186/s13065-025-01549-4 (PMC12224485; doi:10.1186/s13065-025-01549-4)
Supplement: Supplementary file 1 — Supplementary Material 1. [file 13065_2025_1549_MOESM1_ESM.doc]

**Effective elimination of lead from polluted wastewater utilizing a novel nanocomposite derived from zeolite**

**Supplementary Materials**

Table (**S1**): Chemical characteristics of drinking water treatment residuals (WTRs).

| Characteristics^‡^ | Units | WTRs^†^ |
| --- | --- | --- |
| pH |  | 7.45 ± 0.06 |
| EC | dS m^−1^ | 1.67 ± 0.04 |
| O.M^‡^ | g kg^−1^ | 57.00 ± 2.00 |
| KCl-Al | mg kg^−1^ | 28.18 ± 1.03 |
| Olsen-P | mg kg^−1^ | 24.00 ± 2.00 |
| CEC | Cmol(+)kg^−1^ | 34.78 ± 0.34 |
| Total elementsN | g kg^−1^ | 4.20 ± 0.13 |
| P | g kg^−1^ | 1.90 ± 0.15 |
| K | g kg^−1^ | 2.20 ± 0.21 |
| Al | g kg^−1^ | 38.01 ± 0.93 |
| Ni | mg kg^−1^ | 9.40 ± 0.07 |
| Pb | mg kg^−1^ | 76.00 ± 0.17 |
| Cu | mg kg^−1^ | 49.00 ± 0.02 |
| Hg | mg kg^−1^ | 1.04 ± 0.02 |

Table (**S2**): Physical and chemical characteristics of natural zeolite.

| **Physical data** | | **Chemical data*** | |
| --- | --- | --- | --- |
| ITEM | INDEX | ELEMENT | % |
| Appearance | Ivory | SiO_3_ | 78.23 |
| Water solubility | No | Al_2_O_3_ | 11.26 |
| Moisture | ≤10% | K_2_O | 4.62 |
| Density | 0.83 gm/cm^3^ | Fe_2_O_3_ | 1.32 |
| Porosity | 47% | MgO | 1.21 |
| Water absorption | 47% | CaO | 2.86 |
| Surface area | 39 m^2^/gm | Na_2_O | 0.49 |
| Cation Exchange  capacity | 180 meq/100gm |  |  |
| pH | 7.6 |  |  |

Table (**S3**): The chemical analysis of the wastewaters used in the study

|  | Unit | Industrial  effluents | Agricultural drainage |
| --- | --- | --- | --- |
| EC | dSm^-1^ | 769 | 672 |
| pH |  | 9.17 | 9.39 |
| Pb | mgl^-1^ | 3.13 | 5.00 |
| Cd | mgl^-1^ | 2.73 | 5 |
| Na | mgl^-1^ | 57.14 | 5.48 |
| K | mgl^-1^ | 0.94 | 14.10 |


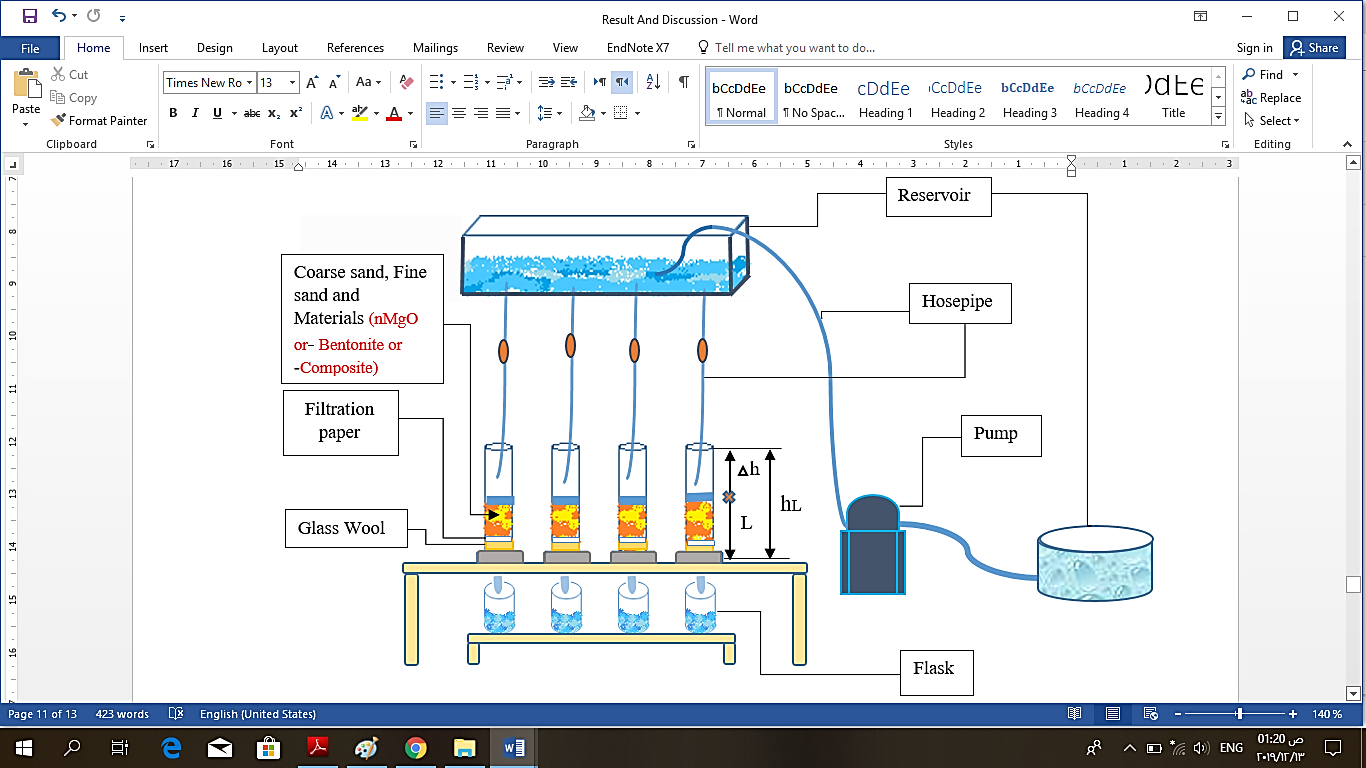


Fig. (**S1**). The schematic diagram of the experimental column.


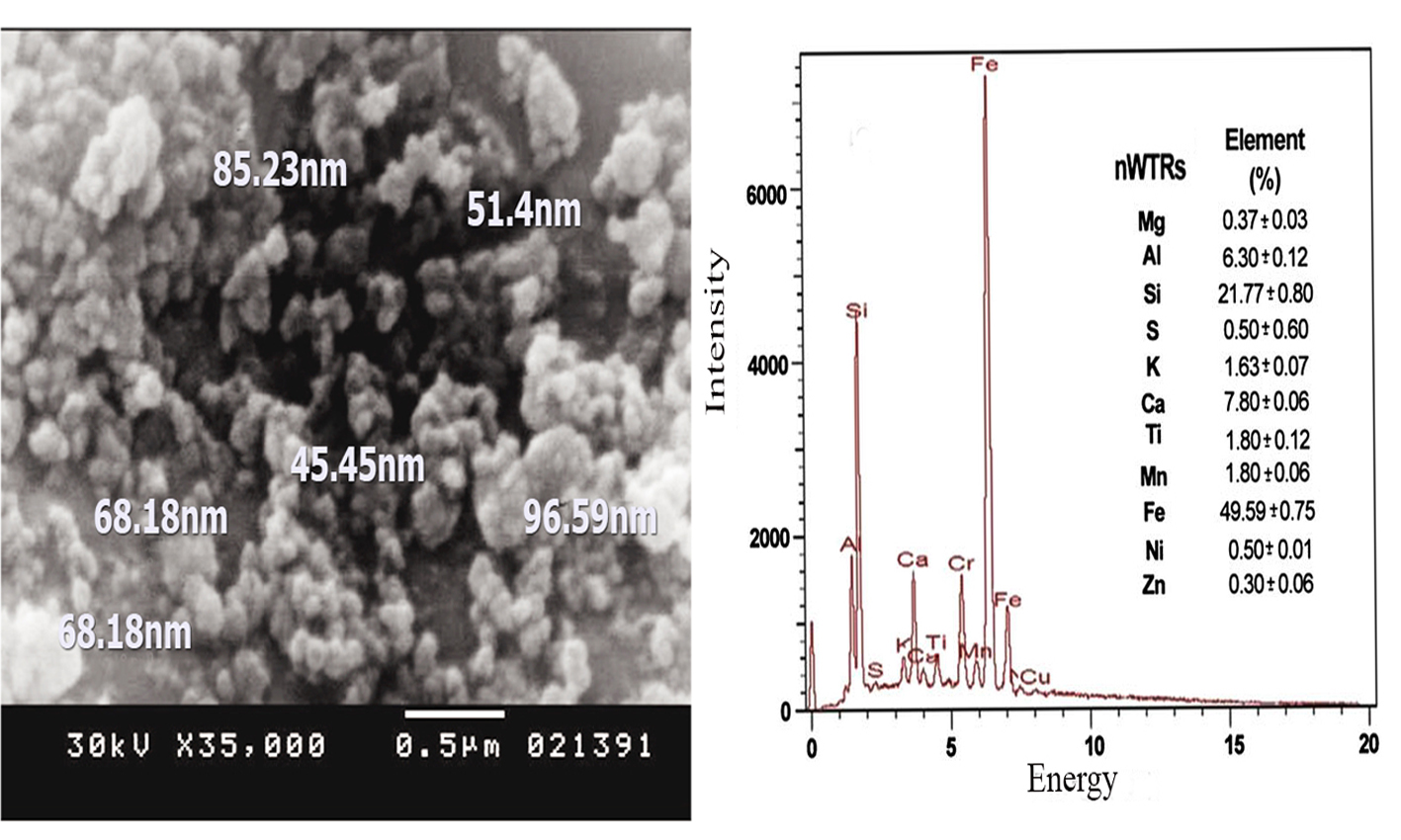

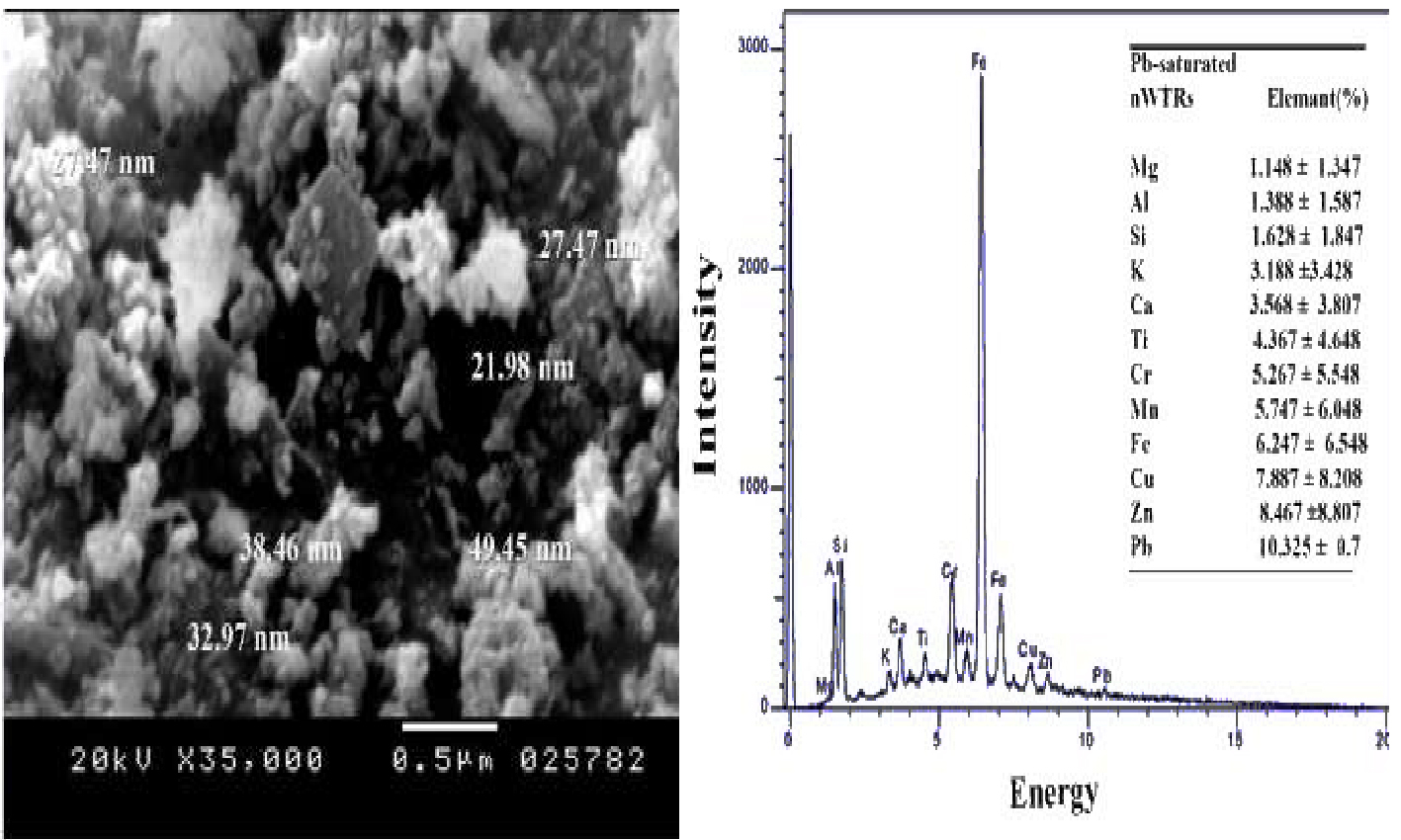


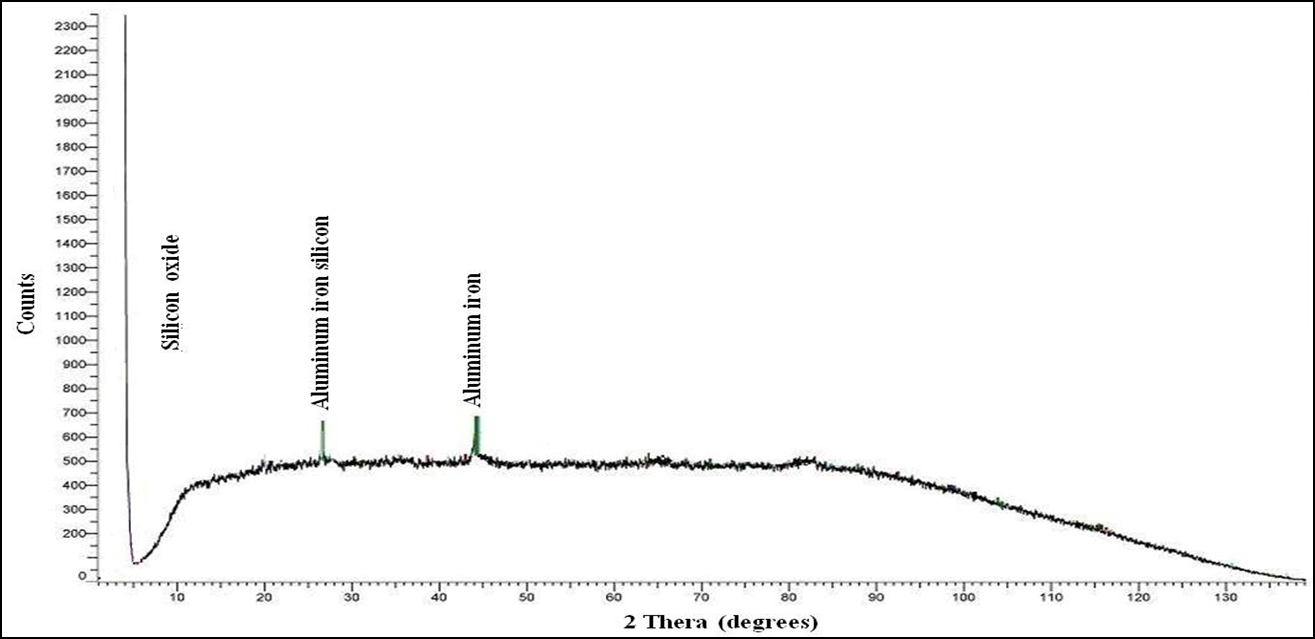


Fig. **(S2**). Scanning electron microscopy (SEM) image and energy-dispersive X-ray (EDX) spectrum of nWTR (above) and Pb-saturated nWTR (middle), and X-ray diffraction (XRD) analyses of nWTR (bottom).

**Thermodynamic parameters**

The thermodynamic parameters including change in the Gibbs free energy (ΔG°, J mol^−1^), enthalpy (ΔH°, J mol^−1^) and entropy (ΔS°, J mol^−1^ K^-1^) were studied to understand the effects of temperature on Pb adsorption process. These parameters were determined using the following equations:

ΔG° = -RT ln K_c_  (1)

Kc = C_qe_/C_S_ (2)

Where:

R = The gas constant [8.314 kJ/ (mol K)]

K_c_ = the equilibrium constant

C_qe_ =The amount of Pb(II) adsorbed on the adsorbent from the solution at equilibrium (mg/L)

C_S_ = The equilibrium concentration of Pb (II) in the solution (mg/ L)

The qe of the Langmuir model was used to obtain C_qe_ and C_S_.

ΔH° and ΔS° were calculated from the plot of ΔG° versus T (**Fig.S3**), by the following equation:

ΔG° = ΔH°−TΔS° (3)

**Fig. S3.** Arrhenius plot of Pb adsorption on nanocomposite(nMgO and Bentonite) (T= 287, 297, and 307 K; pH= 4, 7, and 9; Pb concentrations = 100, 250, 500 and 1000 mgl^-1^).

**The point of zero charge (pH_PZC_)**

The pHpzc is an important feature of the adsorbents that greatly influence the adsorption process as it indicates the net surface charge of the adsorbent in solution. The pH_PZC_ of nanocompsite adsorbent was determined by using the well-known salt addition method reported in literature (Ofomaja and Ho, 2008).

Fig. (**S4**): The pH of point of zero charge (pHzpc) for nanocomposite adsorbent

**Table S4.** The studied adsorption kinetics models and parameters

| **Adsorption kinetics** | **Description** |
| --- | --- |
|  |  |
| **First order**  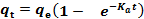 | q or q_t_ = amount of CPF adsorbed at time t . |
|  | q_e_ =amount of CPF adsorbed at equilibrium. |
|  | Ka = apparent adsorbed rate coefficient |
|  | a =constant. |
| **Elovich**  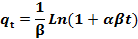 | β = constant related to the extent of surface coverage |
|  | α = the initial adsorbed rate |
|  |  |
| **Parabolic diffusion**  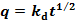 | K_d_ = apparent diffusion rate coefficient |
|  |  |
| **Power function**  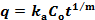 | Ka= apparent adsorbed rate coefficient |
|  | 1/m =constant. |
|  | Co = initial CPF concentration |
|  |  |

**Table S5** The studied adsorption isotherm models and parameters

| **Adsorption isotherms** | **Description** |
| --- | --- |
|  |  |
| **Freundlich**  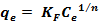 | 𝐾_𝐹_ = constants of Freundlich (the adsorption volume of the adsorbent (. |
|  |  |
|  | 1/n = constants ( intensity of the analytes' sorption). |
|  |  |
| **Langmuir**  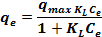 | 𝑞max = maximum adsorption capacity. |
|  |  |
|  | K_L_ =constant of Langmuir ( free energy of adsorption ) . |
|  |  |
| **Temkin**  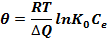 | Δ𝑄 = variation of adsorption energy (−Δ𝐻). |
|  | 𝐾_0_ = constant of Temkin. |
|  | 𝑇 = temperature (K) . |
|  | 𝑅= universal gas constant. |
| **Fowler–Guggenheim(FG)**  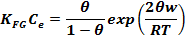 | = interaction energy between adsorbed molecules. |
|  | 𝐾_FG_= constant of Fowler-Guggenheim. |
|  | θ= fractional coverage. |
|  |  |
| **Kiselev**  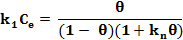 | K**_1=_** constant of Kiselev . |
|  |  |
|  | 𝑘𝑛 = constant (complex formation between adsorbed molecules). |
|  |  |
| **Hill–deBoer**  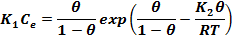 | 𝐾_1_ = constant of Hill-de Boer. |
|  |  |
|  | 𝐾_2_ = constant (interaction between adsorbed molecules) . |
|  |  |
